# Metformin promotes apoptosis of senescent chondrocytes by disrupting the Bcl-xL/Mcl-1–Bak interaction

**DOI:** 10.3389/fphar.2026.1944776

**Published:** 2026-10-28

**Authors:** Biying Feng, Longzhou Liu, Yueyang Lin, Guihao Wu

**Affiliations:** 1 Department of Anesthesiology, The First Affiliated Hospital of Shantou University Medical College, Shantou, China; 2 Department of Pain Management, The Second Affiliated Hospital of Shantou University Medical College, Shantou, China

**Keywords:** apoptosis, Bak, fluorescence resonance energy transfer, metformin, senescent chondrocytes

## Abstract

**Introduction:**

The accumulation of senescent chondrocytes (SEC) beyond the body’s tolerance capacity is a key contributor to osteoarthritis (OA) pathogenesis. Metformin (Met), a first-line oral medication for type 2 diabetes, has shown therapeutic potential in various diseases. This study aims to explore the therapeutic effects and underlying mechanisms of Met in OA.

**Methods:**

Cellular senescence was induced in immortalized human chondrocytes using doxorubicin (Dox), confirmed by β-galactosidase staining and western blotting. The effects of Met on SEC viability, mitochondrial membrane potential, and apoptosis were assessed. Protein expression and interactions were analyzed by western blot and live-cell fluorescence resonance energy transfer (FRET) analysis.

**Results:**

Met promoted death of SEC and reduced mitochondrial membrane potential, inducing caspase- and reactive oxygen species (ROS)-dependent apoptosis. Met upregulated Bak and Bim expression, downregulated Mcl-1, and activated Caspase-3, Caspase-8, and Caspase-9, leading to PARP cleavage. FRET analysis revealed that Met disrupted Mcl-1/Bak and Bcl-xL/Bak binding while facilitating Bim-Bak interaction, promoting Bak dimerization.

**Discussion:**

This study provides the first evidence that Met induces apoptosis of SEC by reducing Mcl-1-Bak and Bcl-xL-Bak interactions while enhancing Bim-Bak binding, suggesting a novel therapeutic strategy for OA by targeting senescent chondrocytes through mitochondrial apoptosis pathways.

## Introduction

Osteoarthritis (OA) is a chronic, irreversible musculoskeletal disorder closely linked to aging, most commonly affecting the hip and knee joints (Safiri et al., 2020). Without proper management, pain and limited mobility often severely impact the quality of life for middle-aged and elderly individuals; OA, along with cancer and cardiovascular diseases, is considered one of the major health threats (Hunter and Bierma-Zeinstra, 2019). Currently, there are no disease-modifying drugs for OA. The aging of joint and cartilage cells is a key pathogenic factor in the development of OA (Loeser et al., 2016). As cell replication reaches a certain threshold, the shortening of telomeres induces physiological aging. Additionally, medications can damage DNA integrity and alter gene expression within cells, leading to pathological aging (Hou et al., 2018; Jeon et al., 2018). Senescent cells release various inflammatory substances, collectively referred to as the senescence-associated secretory phenotype (SASP), which exacerbates pain and functional impairment in OA patients (Jeon et al., 2018; Jeon et al., 2017). As there are currently no disease-modifying drugs for OA (Wang et al., 2019), finding effective treatments to promote cartilage repair and slow disease progression remains a significant challenge.

Metformin (Met) is a first-line oral medication for the treatment of type-2 diabetes, known not only for its ability to lower blood glucose levels but also for its anticancer and anti-aging properties. A substantial body of research, including *in vitro* studies and clinical trials, has shown that Met can slow the progression of OA (Lim et al., 2022; Pan et al., 2025). The current understanding of the potential multifaceted effects of Met in OA primarily includes its possible benefits through the inhibition of inflammation, regulation of autophagy, antagonism of oxidative stress, modulation of adipokine levels, gut microbiota regulation, and reduction of pain levels in OA treatment. It is widely believed that Met exerts its effects mainly through the AMPK-dependent pathway. Met increases the expression of phosphorylated and total AMPK in articular cartilage tissue and limits the development and progression of OA in injury-induced animal models of OA (Li et al., 2020). Met protects against knee OA by reducing chondrocyte apoptosis and catabolism, along with inhibiting the infiltration and pro-inflammatory polarization of synovial macrophages (Li et al., 2023). For senescent cells (SECs), timely clearance is necessary. Studies have shown that Met can promote cell apoptosis (Chen et al., 2021), but it remains unclear whether it can specifically promote SEC apoptosis.

The purpose of this study is to investigate whether Met can eliminate aging chondrocytes and to elucidate its underlying mechanism. This study provides the first confirmation that Met increases the dissociation of Mcl-1 and Bcl-xL from the Bak complex, while promoting the binding of Bim to Bak, thereby facilitating Bak dimerization and inducing apoptosis in SECs.

## Materials and methods

### Materials

Met, Dox, and crystal violet staining solution were purchased from Solarbio (Beijing, China). The JC-1 assay kit (C2003S), senescence β-galactosidase staining kit (C0602), and reactive oxygen species (ROS) detection kit (S0036S) were purchased from Beyotime Biotechnology (Shanghai, China). Anti-P53 (2524), anti-P21 (2947), anti-P16 (23200), anti-PARP (9532), anti-Bak (12105), anti-Bax (5023), anti-Bcl-xL (2764), anti-Mcl-1 (94296), anti-Bim (2933), anti-Puma (4976), and Rabbit monoclonal antibody were purchased from Cell Signaling Technology (Danvers, MA, USA). Anti-β-Actin Mouse monoclonal antibody and Luciferase *Mycoplasma* detection kit were purchased from Transgen Biotech (Beijing, China). Turbofect™ transfection regent and Mito-tracker Deep Red dye were obtained from Thermo Fisher Scientific (Massachusetts, USA). Cell lysis buffer for Western blot and IP (BL509A) was purchased from Biosharp (Hefei, China). Cell counting Kit-8 (CCK-8) and Alexa Fluor® 488 annexin V/Dead Cell Apoptosis Kit were purchased from Dojindo (Kyushu, Japan).

### Constructs

The CFP–YFP dimers, including YFP–G10–CFP and YFP–G40–CFP, were generously provided by Christian Wahl-Schott. Plasmids encoding CFP–Bcl-xL were kindly supplied by A.P. Gilmore. ECFP–Bak (#31501) plasmids were purchased from Addgene. YFP–Bim was synthesized by Gene Create Company. The pCDNA3.1–Mcl-1 (P4841) was designed and synthesized by Miaoling Biotech Company (Wuhan, China). YFP–Bak and CFP–Mcl-1 plasmids were constructed as previously described. All constructs used in this work were confirmed by DNA sequencing and enzyme digestion analysis.

### Cell lines and cell culture

Immortalized human chondrocyte lines (iCell-0092a) were purchased from iCell Bioscience (China); the cells carry the SV40 gene through lentivirus transfection (after transfection, the cells exhibit puromycin-resistance). The cells were cultured in F12 medium containing 10% fetal bovine serum, 100 U/mL penicillin G, and 100 μG/mL streptomycin in a humidified atmosphere at 37° C and 5% CO_2_. All experiments were conducted during the exponential stage of cell growth. Freeze-storage was carried out for the identified cell lines, all of which are in early passaging and kept in the culture medium for less than 3 months. *Mycoplasma* contamination was regularly detected using a luciferase *mycoplasma* detection kit.

### Senescence-associated b-galactosidase assay

The senescence-associated b-galactosidase (SA-b-Gal) assay was performed according to the manufacturer’s instructions for aging-related β-galactosidase (SA-b-Gal) assay (Wu et al., 2022). In summary, cells were cultured in a 12-well plate, washed with PBS, and fixed for 3 min using the fixative solution provided in the kit. Subsequently, after washing with PBS, the plates were incubated overnight with fresh SA-b-Gal staining solution at 37 °C in the absence of CO_2_.

### Cell viability assay

According to the manufacturer’s description, the inhibitory effect of Met on the survival rate of cells was measured using the CCK-8 assay method. In summary, cells were seeded at a density of 1.0 × 10^5^ cells/well in a 96-well plate at a volume of 100 μL, grown at 37 ° C for 24 h, and then treated with different concentrations of Met for 24 h. Subsequently, 10 μL of CCK-8 solution was added to each well, incubated at 37 °C for 1 h, and then the optical density of each well was measured at 570 nm using a microplate reader. All experiments were independently repeated three times. The half maximal inhibitory concentration (IC_50_) value of Met in cells was calculated.

### Colony formation assay

Cells were inoculated (2.0 × 10^5^ cells/mL) into a 6-well plate and treated with Met for 24 h. Both floating and adherent cells were collected and washed twice with PBS. The bottom of the hole is not directly flushed to avoid scattering cells. A measure of 1 mL of 4% paraformaldehyde was added to each well and fixed at room temperature for 20 min. The fixative was gently poured out, wells were gently cleaned once with distilled water, and left to air-dry naturally. A measure of 1 mL of 0.1% crystal violet staining solution was added to each well and stained at room temperature for 15 min. Excess dye was washed off with 33% glacial acetic acid until the background was clean. The samples were thoroughly dried at room temperature, images were taken, and the relative cell coverage was calculated area using ImageJ.

### Flow cytometry analysis of cell apoptosis

Apoptotic cells were identified through Annexin VFITC/PI staining in the Alexa Fluor 488 Annexin V kit. Cells were inoculated (2.0 × 10^5^ cells/mL) into a 6-well plate and treated with Met for 24 h. Annexin V-FITC/PI staining was performed according to the manufacturer’s instructions. Both floating and adherent cells were collected, washed twice with PBS, and resuspended the cells in 1 × binding buffer at a density of 1.0 × 10^6^ cells/mL. It was incubated for 15 min with Alexa Fluor 488 Annexin V conjugate. Necrotic cells were identified with propidium iodide staining. Stained cells were analyzed on a Beckman Coulter CytoFLEX flow cytometer equipped with a 488-nm laser. Alexa Fluor 488 fluorescence was detected in the FITC channel (525/40 nm band-pass filter), and PI fluorescence was detected in the PI channel (585/42 nm band-pass filter). Compensation was performed using single-stained controls (Annexin V-Alexa Fluor 488 only, PI only) and unstained cells; compensation matrix was calculated automatically by CytExpert. Cells were gated on FSC-A/SSC-A to exclude debris, followed by FSC-H/FSC-A gating to exclude aggregates. Quadrant boundaries for Annexin V/PI analysis were established using unstained, Annexin V single-stained, and PI single-stained controls. A minimum of 10,000 events were gated for final analysis.

### ROS-detection flow cytometry analysis

DCFH-DA was diluted with PBS at a ratio of 1:1,000 to achieve a final concentration of 10 mM/L. After cell collection, the cells were suspended in diluted DCFH-DA at a concentration of 1.0 × 10^6^ cells/mL and incubated in a 37 °C cell culture incubator for 20 min. The suspension was inverted and mixed thoroughly every 3 min–5 min. The cells were washed three times with PBS to thoroughly remove DCFH-DA that has not entered the cells. Stained cells were analyzed on a Beckman Coulter CytoFLEX flow cytometer equipped with a 488-nm laser. DCFH-DA fluorescence was detected in the PE channel (585/42 nm band-pass filter). The ROSup included in the reagent kit was used as a positive control.

### Western blotting analysis

To extract the whole protein, cells were collected and washed twice with pre-cooled PBS. Then, the cells were treated with RIPA lysis buffer containing protease inhibitors to obtain cell lysate. Next, the lysate was sonicated and centrifuged at 13,000 rpm for 10 min. The supernatant was retrieved, quantified by BCA protein assay, and stored at −20 °C. Proteins were separated by 12% SDS-PAGE and transferred onto a 0.22-μm polyvinylidene fluoride membrane by electrophoresis. After blocking with 5% skim milk at room temperature for 2 h, the membrane was incubated overnight with rabbit anti human Bak antibody (1:1,000) at 4 °C. After washing with TBST three times, the membrane was incubated with HRP-labeled goat anti rabbit polyclonal antibody (1:10,000) at room temperature for 2 h. After washing with TBST buffer, the immune response band was observed by enhancing chemiluminescence (ECL) of the chemical substrate.

### Protein cross-linking

Cells were lysed in RIPA buffer containing protease inhibitors for 5 min, centrifuged at 13,000 rpm at 4 °C for 10 min, and then the supernatant of the total lysate was collected. An amount of 10 mM BMH or BMOE was added to a final concentration of 0.5 mM. It was incubated at room temperature in the dark for 30 min. Before Western blotting detection, equal volumes of 2-fold reduced SDS-PAGE sample buffer and 5-fold reduced SDS-PAGE sample buffer were added.

### Co-immunoprecipitation

Cells were lysed with protease inhibitors in Western blot and IP cell lysis buffer for 5 min and then centrifuged at 13,000 rpm at 4 °C for 10 min. The supernatant of the total lysate was collected and immunoprecipitated with anti-Bcl-xL or anti-Mcl-1 antibodies. Protein-A/G magnetic beads were collected and washed three times with TBST. Before Western blotting detection, all protein samples were boiled in a standard loading buffer containing 20% loading buffer for 5 min. The subsequent operation was the same as in Western blotting.

### Quantitative FRET imaging

As mentioned earlier, quantitative FRET imaging was performed on a fluorescence microscope (Axio Observer 7, Carl Zeiss, Oberkochen, Germany). In brief, three channel images were captured from the widefield microscopy equipment with a cube with donor excitation and donor emission (DD) for CFP, a cube with acceptor excitation and acceptor emission (AA) for YFP, and a cube with donor excitation and acceptor emission (DA) for FRET. Donor-centric FRET efficiency (*E*_*D*_) and concentration ratio (*R*_*C*_) were measured just as described previously (Chen et al., 2021; Yang et al., 2020).

He-sensitized fluorescence *F*_*C*_ is obtained by subtracting crosstalk, which is obtained by the following formula:

$$F_{C}=I_{DA}-a\left(I_{AA}-cI_{DD}\right)-d\left(I_{DD}-bI_{AA}\right).$$ (1)

Here, *I*_*DA*_ represents the fluorescence intensity detected in the receptor fluorescence detection channel when excited by donor excitation light; *I*_*AA*_ represents the receptor fluorescence intensity detected in the receptor fluorescence detection channel when stimulated by receptor excitation light; *a* and *b* are acceptor bleed-through in the *I*_*DA*_ and *I*_*DD*_ filter sets, and *c* and *d* are donor bleed-through in the *I*_*AA*_ and *I*_*DA*_ filter sets, respectively; *G* is the ratio of the sensitized emission of acceptor to an equivalent quenching of donor, and *k* is the ratio of donor/acceptor fluorescence intensity for equimolar concentrations in the absence of FRET. *G* and *k* can be predetermined using a tandem CFP YFP dimer, just as described previously (Chen et al., 2021; Chen et al., 2025a; Chen et al., 2025b; Yang et al., 2020).

$$a=I_{DA\left(A\right)}/I_{AA\left(A\right)}.$$ (2)

$$b=I_{DD\left(A\right)}/I_{AA\left(A\right)}.$$ (3)

$$c=I_{AA\left(D\right)}/I_{DD\left(D\right)}.$$ (4)

$$d=I_{DA\left(D\right)}/I_{DD\left(D\right)}.$$ (5)

$$G=\frac{F_{C}}{I_{DD}}\cdot \frac{1-E}{E}.$$ (6)

$$k=\frac{I_{DD}+F_{C}/G}{I_{AA}}.$$ (7)

According to Equations 1-7, donor-centric FRET efficiency (*E*_*D*_) and the total concentration ratio (*R*_*C*_) of acceptor to donor were determined as follows:

$$E_{D}=\frac{F_{C}}{F_{C}+GI_{DD}}.$$ (8)

$$R_{C}=\frac{I_{AA}k}{I_{DD}+F_{C}/G}.$$ (9)

### *E*_*D*_ saturation assay

Cells were co-transfected with plasmids encoding CFP tag proteins at 200 ng and plasmids encoding YFP tag proteins at different concentrations (200 ng, 400 ng, 600 ng, and 800 ng, respectively). Generally speaking, only cells with fluorescence signals at least three times higher than the background and less than 60,000 (saturation value of fluorescence signal is 65 535) are selected for analysis. Firstly, spectral permeability parameters were measured from cells expressing CFP (d) or YFP (a) separately according to Equations 2-5. (a = 0.07155, b = 0.000062, c = 0.000011, and d = 0.4785). Secondly, instrument specific calibration constants G = 3.1254 and k = 0.4258 were derived according to Equations 6, 7 using four different donor acceptor dimmers (YFP-G4-CFP, YFP-G10-CFP, YFP-G40-CFP, and YFP-G80-CFP). Then, within the mask of each cell co expressing different plasmids, the total donor concentration [D] and total acceptor concentration [A] were estimated, and the E-FRET efficiency (E_D_) was measured according to Equation 8. The receptor donor concentration ratio (R_c_) can be calculated as [A]/[D] according to Equation 9. For each co-expressed sample, thousands of pixels of *E*_*D*_ and *Rc* were measured and collected to establish an *E*_*D*_–*Rc* map, and the data were boxed by 0.05 *Rc* to obtain the *E*_*D*_–*Rc* FRET curve, which was fitted using the Hill equation to obtain the *E*_*D*_max value. *E*_*D*_ values were distributed into bins of different sizes according to *Rc* and plotted against *Rc*. The saturation binding curve function is:

$$E_{D}=E_{D}\max\times Rc/\left(Kd+Rc\right).$$ (10)

*E*_*D*_max is the maximum *E*_*D*_ corresponding to the saturation of donor binding sites by an acceptor, and *Kd* is the relative equilibrium dissociation constant (Aranovich et al., 2012; Ben-Johny et al., 2016; Butz et al., 2016; Chen et al., 2025a; Chen et al., 2025b).

### Statistical analysis

The *p-*values for data-sets were analyzed by unpaired T-test, and for those that required one-way ANOVA followed by *post hoc* multiple comparison correction, *p* < 0.05 was defined as statistical significance. Statistical and graphic analyses were carried out using Graph Pad Prism 5 and Origin 8.0.

## Results

### Met inhibits the activity of SECs

To confirm whether Dox can induce chondrocyte senescence, we first tested the toxicity of the Dox group to chondrocytes using CCK-8 and obtained an IC_50_ of 3.75 μM (Figure 1A). Then, we screened chondrocytes treated with Dox at a concentration of 50 nM. After 2–3 days, cell proliferation stopped, and the percentage of SA-β-Gal positive cells increased from 2.5% to 79.8% (Figures 1B,C). Next, Western blotting was used to detect aging-related proteins, and it was found that the expression levels of P16, P21, and P53 were increased in Dox-induced senescence chondrocytes (Figures 1D,E), indicating the successful construction of the senescence chondrocyte cell model.

**FIGURE 1:**
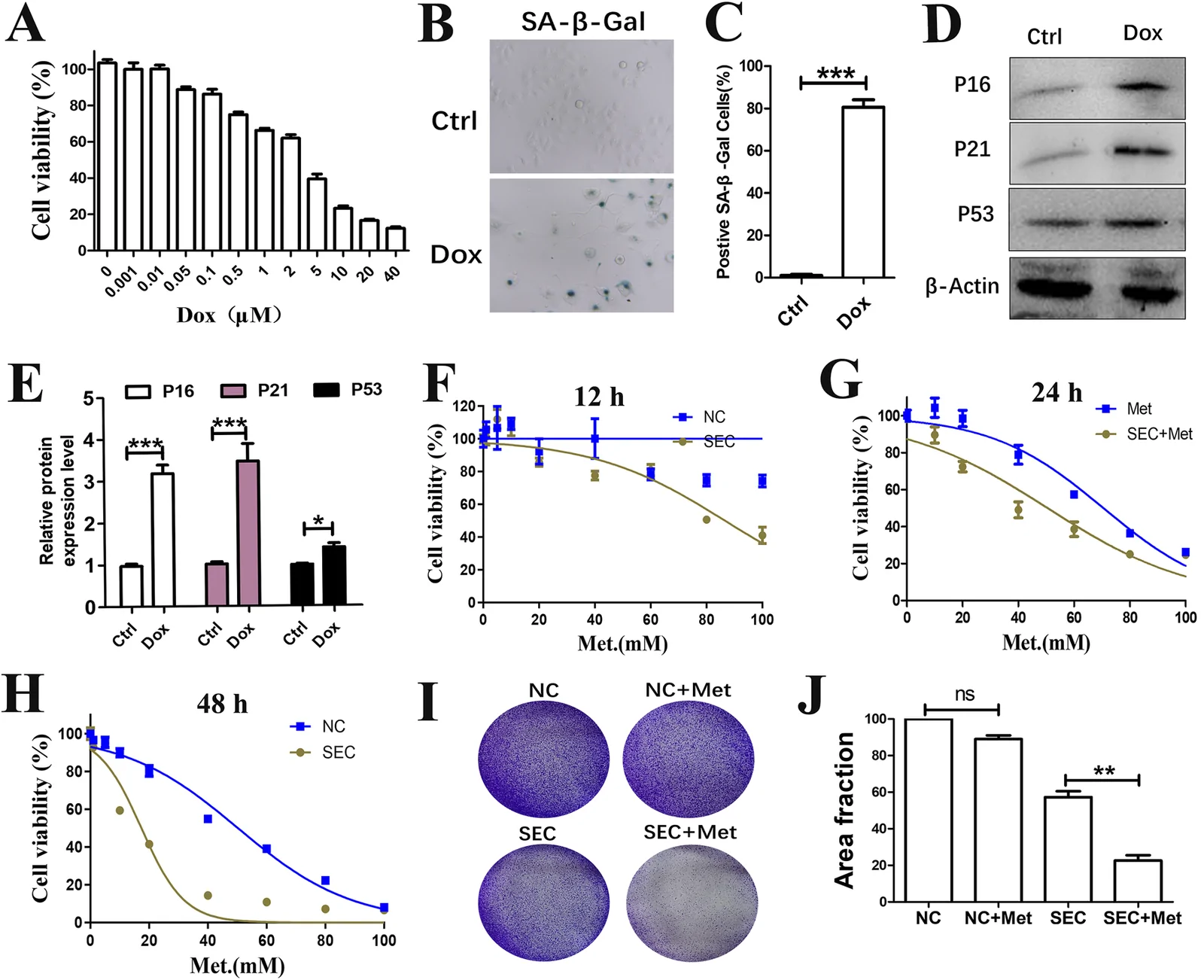
Met inhibits the activity of SECs. **(A)** Dox-induced dose-dependent cytotoxicity measured by CCK-8 assay. **(B)** Representative images of SA-b-Gal staining on samples incubated for 4 days after treatment with 50 nM Dox. **(C)** Percentage of SA-b-Gal-positive cells calculated from at least 500 cells. **(D)** Western blotting analysis on the P-16, P-21, and P-53 expression after 50 nM Dox treatment for 4 days. **(E)** Western blotting statistical plots of P-16, P-21, and P-53. **(F)** CCK-8 method was used to determine the dose-dependent inhibition of SEC activity induced by Met for 12 h. **(G)** CCK-8 method was used to determine the dose-dependent inhibition of SEC activity induced by Met for 24 h. **(H)** CCK-8 method was used to determine the dose-dependent inhibition of SEC activity induced by Met for 48 h. **(I)** Colony formation experiments were conducted to detect the inhibitory effect of Met on SEC activity. **(J)** Statistical chart of colony formation experiment. All data are expressed with the mean ± SEM of three independent experiments. *p < 0.05 and ***p < 0.001 compared with the control group.

To confirm whether Met can inhibit SEC cell activity, we conducted CCK-8 cell activity assays. The results showed that Met had IC_50_ resolutions of 86.85 mM, 40.52 mM, and 17.36 mM for SECs at 12 h, 24 h, and 48 h. Within 100 mM of 12 h, Met showed little toxicity to NC, while the IC_50_ values of 70.54 mM and 50.22 mM for NC (normal chondrocytes) at 24 h and 48 h were observed (Figures 1F–H), indicating that Met inhibited SEC cell activity in a time-dependent and concentration-dependent manner. Next, we proceeded to colony formation experiment, and the results showed that 40 mM Met significantly inhibited SEC activity, but it had no effect on NC activity (Figures 1I,J)

### Met induces caspase- and ROS-dependent apoptosis in SECs

To verify the mode of death induced by Met in SECs, the effect of Met on SEC mitochondrial membrane potential was detected using JC-1 probe, and fluorescence microscopy imaging showed that Met significantly induced the loss of SEC mitochondrial membrane potential, while it had no effect on NC mitochondrial membrane potential at a concentration of 40 mM (Figures 2A,B). To further verify whether Met induces cell apoptosis, Annexin-V/PI staining flow cytometry was used for quantitative analysis. Compared with the control group, Met significantly induced SEC apoptosis after 24 h of Met treatment, while 40 mM Met only induced a small amount of apoptosis in NC (Figure 2C; Supplementary Figure S1A). The time-dependent nature of Met-induced SEC apoptosis is shown in Figure 2D. In addition, flow cytometry analysis showed a significant increase in ROS within SECs, with Met treatment increasing the ROS content within SECs. NAC pretreatment significantly inhibited the increase in ROS caused by SECs and Met (Figure 2E; Supplementary Figure S1B). The activity of caspase detected by Western blotting showed that Met upregulated the expression of caspase-3, caspase-8, and caspase-9 (Figures 2F,G). The use of caspase-inhibitors Z-VAD and NAC (reactive oxygen species inhibitor) significantly inhibited Met-induced SEC death (Figure 2H), indicating that Met induced caspase- and ROS-dependent apoptosis of SECs. PARP cleavage is an important indicator of cell apoptosis and activation of caspase-3, and Met-induced PARP cleavage further confirms the induction of SEC cell apoptosis (Figures 2I,J).

**FIGURE 2:**
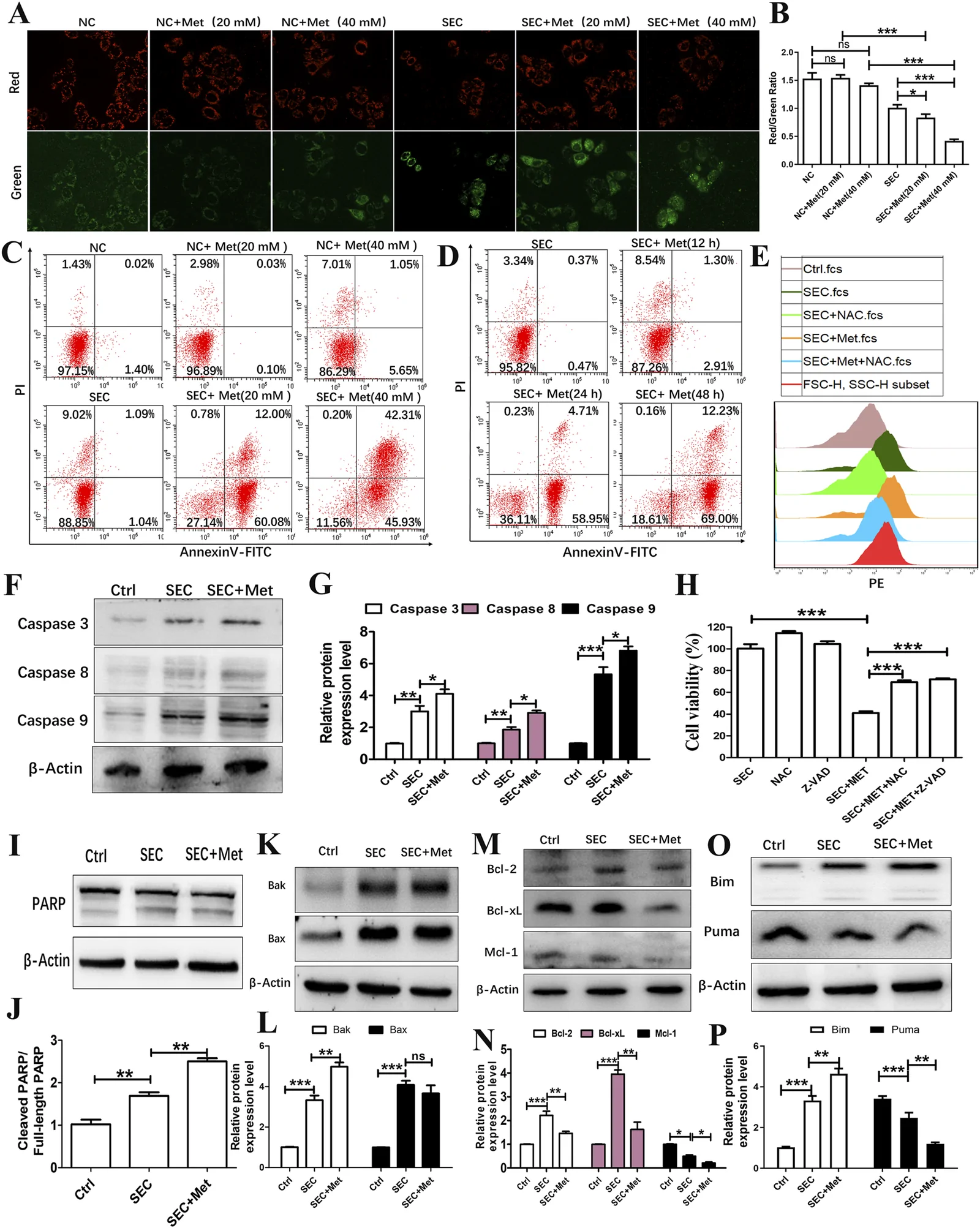
Met induces caspase- and ROS-dependent apoptosis in SECs. **(A)** Met-induced decrease in mitochondrial membrane potential. **(B)** Statistical chart of Met-induced decrease in mitochondrial membrane potential (n = 100). **(C)** Quantitatively analyzed percentage of apoptotic cells in Annexin-V/PI-stained cells using flow cytometry. **(D)** Time-dependence of Met-induced SEC apoptosis. **(E)** Met induced a significantly increased intracellular ROS. **(F)** Western blotting analysis of caspase-3, caspase-8, and caspase-9 expression after 24 h of treatment with 30 mM Met. **(G)** Western blotting statistical plots of caspase-3, caspase-8, and caspase-9. **(H)** NAC and Z-VAD significantly inhibited Met-induced cytotoxicity. **(I)** Western blotting analysis on the Met-induced PARP cleavage. **(J)** Western blotting statistical plots of PARP cleavage. **(K)** Western blotting analysis of Bak and Bax after 24 h of treatment with 30 mM Met. **(L)** Western blotting statistical plots of Bak and Bax. **(M)** Western blotting analysis of Bcl-2, Bcl-xL, and Mcl-1 after 24 h of treatment with 30 mM Met. **(N)** Western blotting statistical plots of Bcl-2, Bcl-xL and Mcl-1. **(O)** Western blotting analysis of Bim and Puma after 24 h of treatment with 30 mM Met. **(P)** Western blotting statistical plots of Bim and Puma. All data are expressed with the mean ± SEM of three independent experiments. *p < 0.05, **p < 0.01, and ***p < 0.001 compared with the control group.

Western blotting analysis showed that Met upregulated Bak and Bax expression in SECs (Figures 2K,L), indicating that Bak and Bax may be key proteins in Met-induced SEC apoptosis. Anti-apoptotic proteins (Bcl-2, Bcl-xL, and Mcl-1) inhibit apoptosis by binding to pro-apoptotic proteins. Western blotting analysis showed that Met reduced the expression of Mcl-1, Bcl-xL, and Bcl-2 in SECs compared to that in untreated SECs (Figures 2M,N). BH3 only protein is also a key protein for Bak activation to promote apoptosis. Met significantly induces the expression of Bim but downregulates the expression of Puma (Figures 2O,P).

### Met unlocks the binding of Mcl-1/Bcl-xL and Bak in SECs

Anti-apoptotic proteins (Bcl-2, Bcl-xL, and Mcl-1) play an important role in the process of cell apoptosis by binding and separating with pro-apoptotic proteins (Bak and Bax) (García-Sáez, 2012; Singh et al., 2019). Quantitative FRET measurements were performed on SEC live cells co-expressing CFP–Bcl-xL and YFP–Bak (Figure 3A), and Figure 3B shows the corresponding *E*_*D*_–*Rc* plots from 89 cells. When *Rc* is greater than 1.5, the two binding curves are saturated, and the *E*_*D*max_ of 0.391 ± 0.021 in SECs is greater than that of 0.203 ± 0.016 in control cells according to Equation 10, indicating an increase in the binding of Bcl-xL and Bak in SECs. In addition, the *E*_*D*max_ of 0.142 ± 0.012 in cells treated with Met was lower than that in the control group and the SEC group according to Equation 10, indicating that Met-treatment is associated with reduced Mcl-1–Bak interactions (Figures 3B,C). Co-IP analysis showed that Met released the connection between Bcl-xL and Bak, which is consistent with the FRET results (Figure 3D).

**FIGURE 3:**
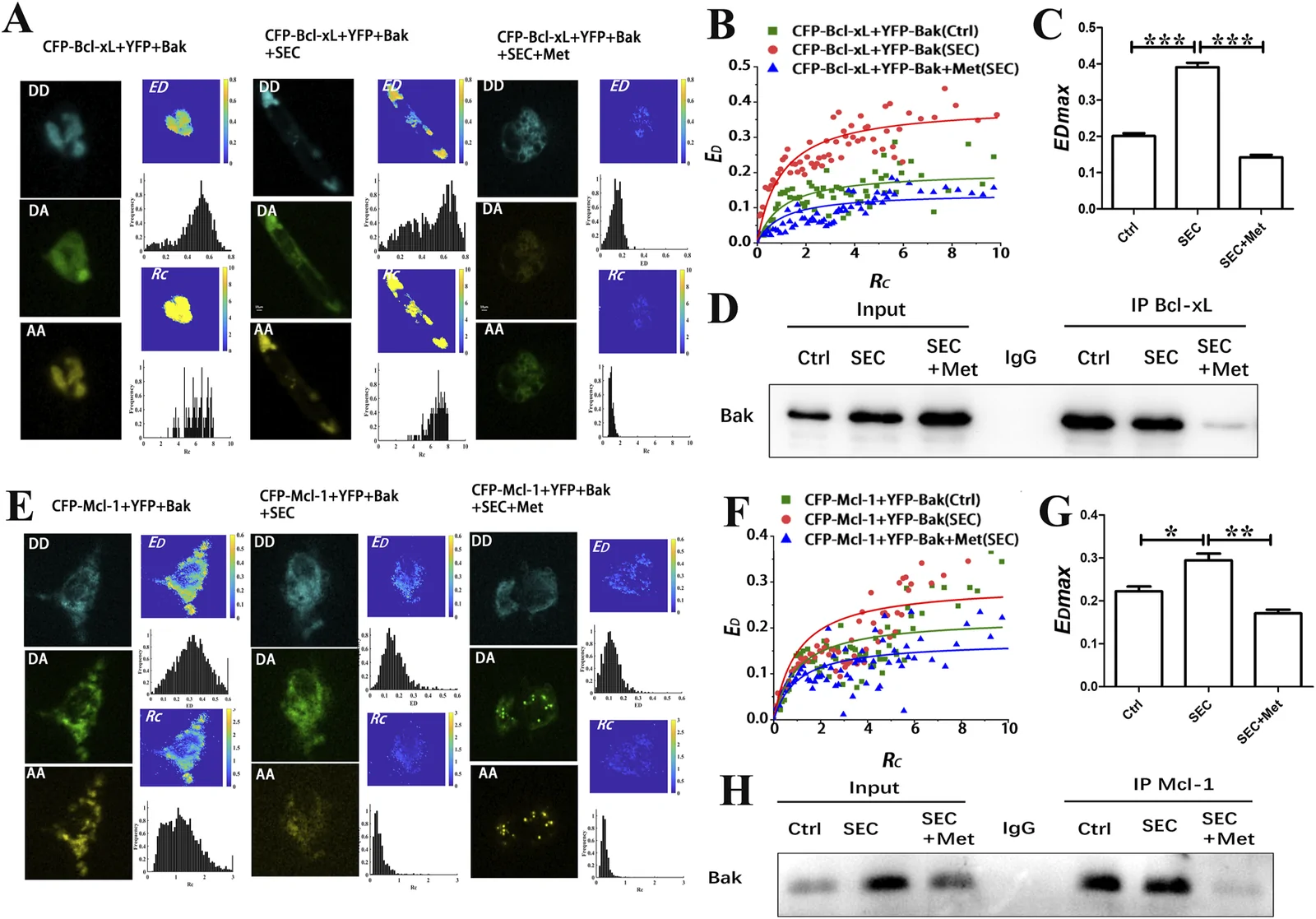
Met unlocks the binding of Mcl-1/Bcl-xL and Bak in SEC. **(A)** Three fluorescence images (DD, DA, and AA) and the corresponding pseudo-color *E*_*D*_ and *Rc* images of living cells co-expressing CFP–Bcl-xL and YFP–Bak. **(B)** *E*_*D*_ *–Rc* plots from co-expressing CFP–Bcl-xL and YFP–Bak. **(C)** Statistical plots of *E*_*D*max_. **(D)** Co-IP analysis on the binding of Bcl-xL to Bak. **(E)** Three fluorescence images (DD, DA, and AA) and the corresponding pseudo-color *E*_*D*_ and *Rc* images of living cells co-expressing CFP–Mcl-1 and YFP–Bak. **(F)** *E*_*D*_ *–Rc* plots from co-expressing CFP–Mcl-1 and YFP–Bak. **(G)** Statistical plots of *E*_*D*max_. **(H)** Co-IP analysis on the binding of Mcl-1 to Bak. All data are expressed with the mean ± SEM of three independent experiments. *p < 0.05, **p < 0.01, and ***p < 0.001 compared with the control group.

Similarly, quantitative FRET measurements were performed on SEC live cells co-expressing CFP–Mcl-1 and YFP–Bak. Figure 3E shows the corresponding *E*_*D*_–*Rc* plots from 103 cells (Figure 3E). When *Rc* is greater than two, the two binding curves are saturated, and the *E*_*D*max_ of 0.295 ± 0.027 in SECs is greater than the *E*_*D*max_ of 0.222 ± 0.020 in control cells according to Equation 10, indicating an increase in the binding of Mcl-1 and Bak in SECs. In addition, the *E*_*D*max_ of 0.171 ± 0.015 in cells treated with Met was significantly lower than that in the control group and SEC group according to Equation 10 (Figures 3F,G), indicating that Met-treatment is associated with reduced Mcl-1–Bak interactions. Co-IP analysis showed that Met released the binding of Mcl-1 and Bak (Figure 3H), which is consistent with FRET results.

### Met promotes the binding of Bim and Bak and promotes Bak dimerization in SECs

BH3-only proteins including Bim, tBid, and Puma can directly activate Bak. In order to evaluate the effect of Met on the binding of Bim and Bak in SECs, quantitative FRET was used to measure the *E*_*D*_–*Rc* curves between CFP–Bim and YFP–Bak in 93 cells (Figure 4A). When *Rc* is greater than two, the two binding curves are saturated. The *E*_*D*max_ in SECs is 0.365 ± 0.0301 according to Equation 10, while that in control cells is 0.276 ± 0.025 according to Equation 10, indicating that aging promotes the binding of Bim and Bak. The *E*_*D*max_ in cells treated with Met is 0.410 ± 0.034 according to Equation 10, which is significantly higher than that in control cells and SECs (Figures 4B,C), indicating that Met promotes the binding of Bim and Bak, which may activate Bak.

**FIGURE 4:**
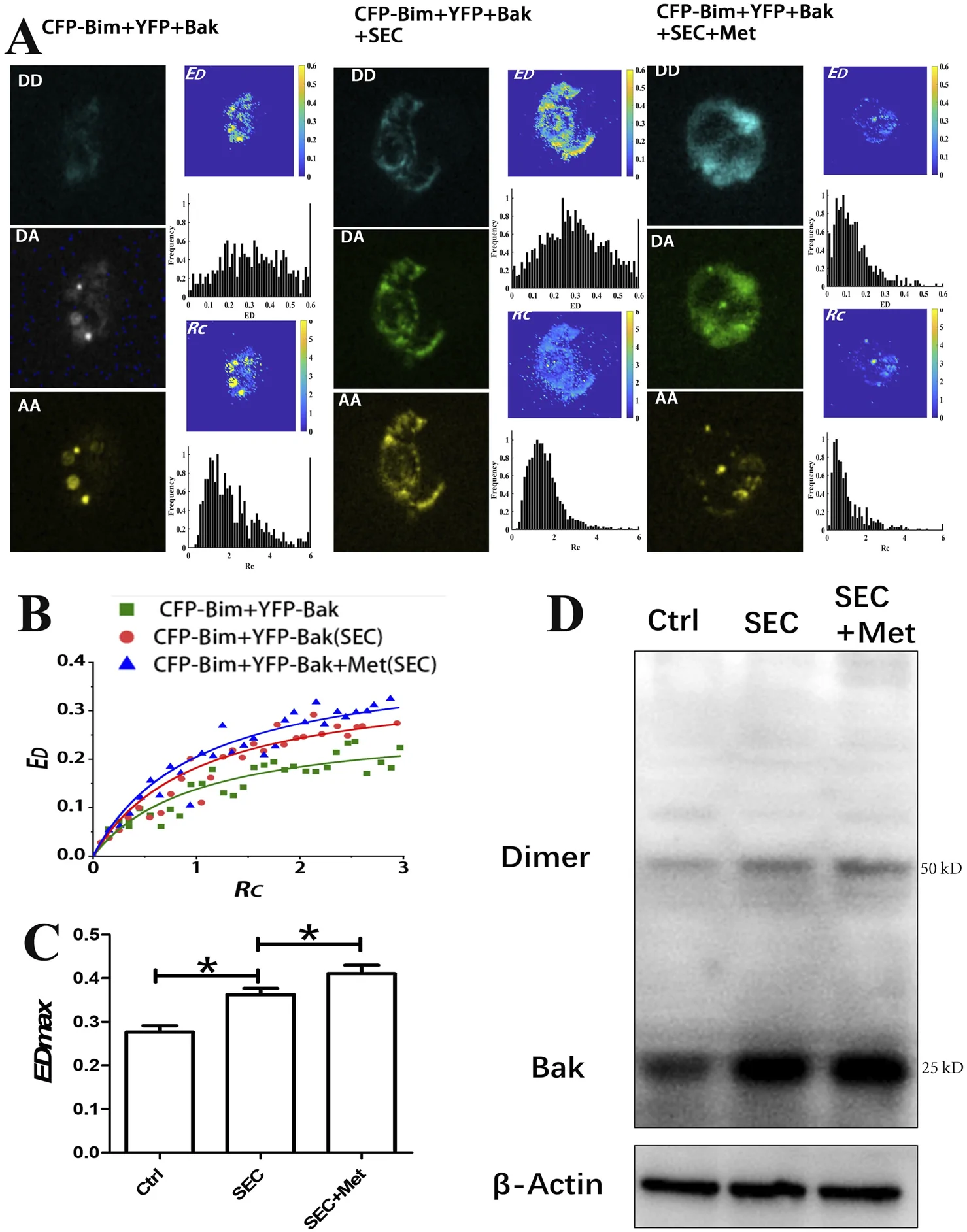
Met promotes the binding of Bim and Bak and promotes Bak homo-oligomerization in SECs. **(A)** Three fluorescence images (DD, DA, and AA) and the corresponding pseudo-color *E*_*D*_ and *Rc* images of living cells co-expressing CFP–Bim and YFP–Bak. **(B)** *E*_*D*_ *–Rc* plots from co-expressing CFP–Bim and YFP–Bak. **(C)** Statistical plots of *E*_*D*max_. **(D)** Effect of Met on Bak homooligomerization by protein crosslinking analysis. All data are expressed with the mean ± SEM of three independent experiments. *p < 0.05 compared with the control group.

Bax and Bak, as the main effectors of the Bcl-2 family, undergo homologous oligomerization on mitochondria, leading to the occurrence of cell apoptosis. The protein cross-linking experiment showed that the dimer of Bak increased in SECs, and Met-treatment further promoted Bak dimerization (Figure 4D), indicating that Met promotes the dimerization of Bak in SECs.

## Discussion

OA is the most common degenerative joint disease, and its incidence rate increases significantly with age. In recent years, the key role of SECs in the development of OA has been increasingly recognized; When SECs exceed the body’s clearance capacity, they will continue to secrete inflammatory factors and matrix-degrading enzymes, accelerating the destruction of joint cartilage. Eliminating excessive accumulation of SECs has emerged as a potential strategy for preventing and treating OA. This study reveals, for the first time, that Met mainly induces caspase- and ROS-dependent cell apoptosis through the intrinsic pathway mediated by Bak, reducing binding of Mcl-1 and Bcl-xL to Bak. BH-3 protein Bim serves as a key initiator to activate Bak, promote Bak dimerization, and induce SEC apoptosis. While our CCK-8 data indicate that Met preferentially reduces viability in SECs compared to NCs at equivalent concentrations, the mechanistic experiments in this study were performed exclusively on senescent cells. These findings provide proof-of-concept mechanistic evidence for Met’s pro-apoptotic effects in SECs.

Met-induced SEC-death conforms to the biochemical characteristics of classical cell apoptosis. We observed a significant loss of SEC mitochondrial membrane potential after Met treatment, accompanied by the activation of caspase-3, caspase-8, and caspase-9 and the cleavage of PARP. The pan-caspase inhibitor Z-VAD effectively blocked this process, confirming that Met clears SECs by activating the caspase-dependent apoptotic pathway. It is worth noting that the activation of caspase-8 indicates a possible overlap between exogenous and endogenous apoptotic pathways. In addition, Met significantly increased the level of ROS within SECs, and the ROS scavenger NAC significantly reduced cell death, indicating that oxidative stress plays an important auxiliary or amplifying role in this process. This is consistent with reports that Met induces oxidative stress and promotes apoptosis in other cell types, indicating that ROS may be an upstream or parallel signal of Met’s pro apoptotic effect.

This study found that Met can reshape the expression profile of the Bcl-2 family in SECs: downregulating anti apoptotic proteins Mcl-1, Bcl-xL, and Bcl-2, while upregulating pro-apoptotic effector proteins Bak, Bax, and BH3-only protein Bim. This imbalance of “pro-apoptotic/anti-apoptotic” creates conditions for mitochondrial outer membrane permeabilization (MOMP). Our observations indicate that the expression levels of anti-apoptotic proteins Bcl-xL and Bcl-2 in SECs are elevated (Figures 2K,L). This is consistent with previous findings (Rochette and Brash, 2008; Wang, 1995). At the same time, we also found that the expression levels of SEC pro-apoptotic proteins Bak and Bax increased, but the cells did not die, which may be related to the increased expression levels of Bcl-xL and Bcl-2. However, changes in protein expression alone are not sufficient to fully explain the initiation of apoptosis. The physical interactions between proteins are the key limiting step in determining cell fate.

In the apoptosis regulatory network, the interaction of Bcl-2 family proteins determines cell fate. The binding between anti-apoptotic proteins and pro-apoptotic proteins in the Bcl-2 family can inhibit pro-apoptotic function, and Bcl-xL and Bcl-2 can effectively inhibit Bak activation (Singh et al., 2019; Youle and Strasser, 2008). Using quantitative live-cell FRET technology, this study elucidated the core mechanism of Met at the molecular interaction level. The results showed that in Dox-induced SECs, the binding affinity between anti-apoptotic proteins Bcl-xL and Mcl-1 and effector protein Bak was significantly enhanced, forming a tighter “isolated” state, which may be an important mechanism for aging cells to resist apoptosis and sustain survival. After Met treatment, FRET *E*_*D*max_ values for Bcl-xL/Bak and Mcl-1/Bak decreased significantly, indicating reduced proximity between these protein pairs. Co-IP experiments showed diminished Bak co-precipitation with Bcl-xL and Mcl-1. However, because Met concurrently downregulates Mcl-1/Bcl-xL expression and upregulates Bak, these changes may reflect altered protein abundance rather than genuine complex dissociation. Whether this represents a true unlocking of Bak or simply reduced availability of anti-apoptotic binding partners requires further investigation with quantitative normalized Co-IP and functional rescue experiments. This unlocking effect releases Bak’s pro-apoptotic activity. The key execution of apoptosis is BH3-only protein: Bim, Bif, and Puma, which bind and activate pro-apoptotic Bak or Bax (Minet et al., 2006; Singh et al., 2019). Met significantly enhanced the binding of BH3-only protein Bim to Bak and promoted the dimerization of Bak. Bak dimerization is the direct cause of the formation of mitochondrial outer membrane pores and the release of pro-apoptotic factors. Therefore, Met drives Bak dimerization and cell apoptosis through a “dual approach”—both relieving the inhibition of Bak by anti-apoptotic proteins and enhancing the direct activation of Bak by Bim. This mechanism is highly consistent with the classical theory that Bcl-2 family proteins finely regulate apoptosis through binding and separation. The present study establishes that Met enhances Bim–Bak association and Bak oligomerization in SECs, consistent with a Bim-driven Bak-activation model. However, definitive proof that Bim is required for Met-induced Bak oligomerization and apoptosis awaits genetic intervention studies (siRNA/shRNA knockdown or CRISPR knockout of Bim and/or Bak). Similarly, the potential contribution of Bax—which is also upregulated by Met—cannot be excluded without Bax-specific silencing experiments. Time-course analyses of Bak conformational activation (using N-terminal exposure antibodies) and cytochrome-c release would further delineate the proposed sequence of events. These experiments represent critical priorities for follow-up studies.

Met, as a first-line drug for type-2 diabetes, has long-term clinical experience and a good safety record. The findings of this study indicate that Met may delay the degenerative process of OA by clearing excessive accumulation of SECs in the joint cavity. Some new drugs that can remove SECs have been under development, such as dasatinib, quercetin, and pemeramide (Childs et al., 2017). In addition, some studies have shown that A1331852 and ABT-263 can selectively eliminate senescent cells (Pan et al., 2017; Wu et al., 2022; Yosef et al., 2016). However, ABT-263 is a Bcl-2 family anti-apoptotic protein inhibitor, which can inhibit the functions of Bcl-2, Bcl-xL, Bcl-w, and it has no effect on Mcl-1. However, its clinical use has a large side-effect, which can lead to thrombocytopenia and neutropenia (Wilson et al., 2010). Met is a type-2 diabetes drug that has been used clinically for many years (Sanchez-Rangel and Inzucchi, 2017), and some research studies have also shown that Met has multiple effects, such as anti-aging, anti-tumor, and weight loss, among others (Brown et al., 2020; Day et al., 2019; Guarente et al., 2024; Wu et al., 2019; Yerevanian and Soukas, 2019). These applications demonstrate that Met has high security. However, this study still has certain limitations. First, the experiment was conducted only in immortalized human chondrocyte lines, using Dox-induced aging models. In the future, it will need to be validated in primary chondrocytes and other models such as replicative aging and stress-induced aging. Second, the effective concentration of Met *in vitro* is relatively high (30 mM–40 mM), and the concentration used in *in vitro* studies is higher than typical therapeutic plasma levels. We observed a bimodal reaction pattern using a wider concentration range (0.1 mM–100 mM) in SECs and NCs at 24 and 48 h: SECs exhibited significant selective cytotoxicity within 24 h at a concentration of 20 mM, and the survival rate of SECs was significantly reduced, while the survival rate of NC remained above 90%. When the concentration is 10 mM within 48 h, the cell-survival rate of SECs is only 70%, while the survival rate of NCs remains above 90%. At 20 mM, the apoptosis rate of SECs was [72.84%], while that of NCs was [3.11%]. We believe that high-concentration data (≥20 mM) may reflect non-specific stress-responses. Whether this dose can be achieved and is safe for local or systemic joint administration *in vivo* still needs to be further evaluated through animal OA models (such as DMM or ACLT models). While this study is focused on AMPK-independent Bcl-2 family mechanisms, the canonical AMPK pathway was not examined. AMPK activation can exert both cytoprotective and pro-apoptotic effects depending on the context, dose, and cell type. At suprapharmacological concentrations, non-specific AMPK hyperactivation could contribute to cellular stress. Future studies must determine whether AMPK inhibition attenuates or exacerbates metformin-induced SEC apoptosis and whether AMPK and Bcl-2 family pathways converge or operate independently in this context.

This study has a limitation regarding selectivity validation. While CCK-8 assays demonstrated differential toxicity between senescent and NCs, all mechanistic apoptosis assays (caspase activation, PARP cleavage, mitochondrial membrane potential, Bak dimerization, and FRET analysis) were conducted exclusively in SECs. Parallel normal chondrocyte controls were not included in these experiments. Consequently, we cannot definitively conclude that the molecular mechanisms identified here are unique to senescent cells. Future studies must directly compare metformin-induced apoptotic signaling in normal versus senescent chondrocytes to confirm senolytic selectivity. While the concentrations used in this *in vitro* study exceed typical plasma therapeutic levels, they serve to uncover the molecular mechanism of metformin-induced SEC apoptosis. Clinical translation would require validation in animal OA models with local delivery systems and optimization of dose formulations to achieve therapeutic joint concentrations without systemic toxicity.

In summary, in this study, we elucidated that Met induces Bak dimerization and caspase/ROS-dependent apoptosis by breaking the inhibitory binding of Bcl-xL/Mcl-1–Bak and promoting the activation binding of Bim–Bak, thereby achieving the clearance of SECs. This discovery not only expands the understanding of the pharmacological effects of Met but also provides a new theoretical basis and potential intervention targets for targeted clearance of aging cells in the treatment of OA.

## Data Availability

The original contributions presented in the study are included in the article/Supplementary Material; further inquiries can be directed to the corresponding author.
